# Drunk Driver Detection Using Multiple Non-Invasive Biosignals

**DOI:** 10.3390/s25051281

**Published:** 2025-02-20

**Authors:** Sang Hyuk Kim, Hyo Won Son, Tae Mu Lee, Hyun Jae Baek

**Affiliations:** Department of Biomedical Engineering, College of Medical Sciences, Soonchunhyang University, Asan 31537, Republic of Korea; sanghyuk.kim@sch.ac.kr (S.H.K.); gydnjs0192@sch.ac.kr (H.W.S.); rouon1594@sch.ac.kr (T.M.L.)

**Keywords:** drunk driving detection, non-invasive biological signal monitoring, machine learning

## Abstract

This study aims to decrease the number of drunk drivers, a significant social problem. Traditional methods to measure alcohol intake include blood alcohol concentration (BAC) and breath alcohol concentration (BrAC) tests. While BAC testing requires blood samples and is impractical, BrAC testing is commonly used in drunk driving enforcement. In this study, the multiple biological signals of electrocardiogram (ECG), photoplethysmogram (PPG), and electrodermal activity (EDA) were collected non-invasively and with minimal driver restraint in a driving simulator. Data were collected from 10 participants for approximately 10 min at BrAC levels of 0.00%, 0.03%, and 0.08%, which align with the latest Korean drunk driving standards. The collected data underwent frequency filtering and were segmented into 30 s intervals with a 10 s overlap to extract heart rate variability (HRV) and pulse arrival time (PAT). Using more than 10 machine learning algorithms, the classification accuracy reached 88%. The results indicate that it is possible to classify a driver’s level of intoxication using only non-invasive biological signals within a short period of about 30 s, potentially aiding in the prevention of drunk driving.

## 1. Introduction

In addition to drivers’ violations of traffic laws and driving habits, human factors such as drowsiness, fatigue, and stress account for the majority of traffic accidents. Drunk driving reduces a driver’s field of vision and impairs their judgment, increasing the likelihood of traffic accidents. Drunk driving is also reported to be responsible for more than 40% of all traffic accidents [1,2,3,4,5]. Drunk driving remains a pervasive and deadly issue worldwide, with significant social and economic consequences [6,7,8,9]. Despite stringent legal frameworks and widespread public awareness campaigns, alcohol-impaired driving continues to result in a substantial number of traffic accidents, injuries, and fatalities [10,11,12,13]. The persistence of this problem underscores the need for innovative approaches to detect and prevent intoxicated driving before it leads to harm [14]. Current methods for detecting alcohol intoxication, such as blood alcohol concentration (BAC) and breath alcohol concentration (BrAC) tests, are effective but have limitations [15]. BAC testing, while highly accurate, is invasive and impractical for regular or on-the-spot use. BrAC testing, more commonly used in law enforcement, offers a non-invasive alternative but can be affected by external factors such as residual mouth alcohol and environmental conditions, leading to potential inaccuracies [16]. Moreover, these methods typically require active cooperation from the driver and are often applied reactively, after a driver is already on the road, rather than as a preventive measure. In contrast, non-contact biometric signals present a promising avenue for more proactive, continuous monitoring of intoxication levels [17,18,19,20,21,22,23,24,25].

Biometric signals, such as those of electrocardiograms (ECGs), photoplethysmograms (PPGs), and electrodermal activity (EDA), are of particular interest because they can be monitored in real time, continuously, and without interfering with the driving experience. These signals reflect various physiological changes that occur with alcohol consumption, including alterations in heart rate variability (HRV), blood flow, and skin conductivity. By analyzing these signals, it may be possible to infer a driver’s intoxication level without the need for invasive testing [26,27]. The hypothesis that drinking can be predicted using non-contact biometric signals is grounded in the understanding that alcohol affects the autonomic nervous system, leading to measurable changes in physiological functions [28]. For example, alcohol consumption typically reduces HRV, as it depresses the parasympathetic nervous system while stimulating the sympathetic nervous system. Similarly, alcohol can influence PPG readings by altering blood vessel dilation and blood flow dynamics. EDA, which measures skin conductance, is sensitive to changes in emotional and physiological arousal, both of which can be affected by alcohol. By capturing and analyzing these biometric signals, it may be possible to develop a robust model that can accurately predict alcohol intoxication [26].

An important aspect of this approach is the speed of detecting driver impairment. In real-world scenarios, especially in automotive safety applications, it is essential to quickly identify a driver’s state of intoxication or drowsiness to prevent accidents [29,30]. First, the earlier intoxication is detected, the sooner preventive measures can be implemented, such as alerting the driver, reducing vehicle speed, or preventing vehicle use to prevent an accident. This proactive approach is crucial for minimizing the risks associated with drunk driving. Second, the practical application of such technology demands that the detection process be simple and unobtrusive, integrating smoothly into the driving experience without causing distraction or lengthy interruptions [31,32,33,34,35]. Among previous studies on the recommended duration for HRV analysis, second-by-second HRV analysis was validated using advanced statistical methods, with recommendations to record ECG for a minimum of 10 s for RMSSD and 30 s for SDNN [36]. This timeframe allows the collection and analysis of sufficient biometric data to make a confident determination of intoxication while still being short enough to enable immediate intervention if necessary [37].

Moreover, as vehicles become increasingly autonomous and equipped with advanced safety features, the ability to monitor and respond to a driver’s physiological state in real time becomes even more critical. Autonomous vehicles, which rely on a combination of sensors and algorithms to operate safely, could benefit from integrating biometric monitoring systems to ensure that human drivers or passengers are in an optimal state to take control if required [21]. Rapid detection of intoxication would allow these systems to make more informed decisions, further enhancing overall road safety.

## 2. Materials and Methods

### 2.1. Biosignal Data Collection

In our experiment, ECG, PPG, and EDA data were collected from a driver using a driving simulator (YESA-HW2, Young-il Education System, Seoul, Republic of Korea) equipped with three connected monitors. To effectively gather biosignal data from drivers, a nonintrusive measurement technique that allows natural data collection without requiring driver active cooperation or interfering with driving behavior is essential, and research in this area is active [38,39]. In our previous study, a method for nonintrusive measurement of ECG, PPG, and EDR using sensors embedded in the steering wheel, respiratory signals via seat belts, and ECG signals from capacitively coupled electrodes in the vehicle seat was developed [40].

In the present study, capacitively coupled ECG electrodes were implemented on the driver’s seat of the driving simulator to nonintrusively collect ECG signals during driving. Simultaneously, PPG and EDA data were collected using commercially available biosignal measurement devices (OXY100E and EDA100C, respectively; Biopac Systems Inc., Goleta, CA, USA). Although these were not non-constrained measurements, it is expected that non-constrained measurements can be achieved by integrating sensors into the steering wheel, as demonstrated in previous studies [41,42,43]. All signals were digitized at a sampling rate of 2000 Hz using the BIOPAC MP160 system. To achieve non-contact ECG signal measurement through insulating materials such as clothing, the concept of displacement current was employed. The insulating properties of clothing create a relatively high impedance between the sensor and the skin. Therefore, a high-input impedance amplifier (OPA196, input impedance: 10 TΩ, Texas Instruments, Dallas, TX, USA) was used as the active electrode in each sensor to convert the displacement current passing through the clothing into a voltage.

**Figure 1:**
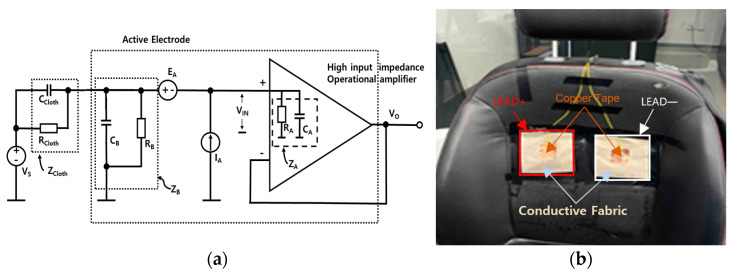
(**a**) Schematic diagram of the simple principle of a capacitance electrode. (**b**) Photograph of an example application on a real driver’s seat, in which a conductive cloth is attached to a seat and connected with a capacitance electrode to obtain an ECG with everyday clothes.

Figure 1a shows a diagram of the capacitive electrode, including the electrode–body interface model Z_Cloth_. The inherent noise of the operational amplifier is modeled using a voltage noise source (E_A_) and a current noise source (I_A_). The resistor R_B_ serves to provide bias current for the operational amplifier. The total capacitance between the input (including the electrode surface and input circuitry) and the circuit ground is denoted as C_B_. Additionally, R_A_ and C_A_ represent the input resistance and capacitance of the operational amplifier, respectively. The capacitively coupled electrode essentially consists of an AC-coupled voltage follower. The gain of the circuit is defined as:

$$G_{s}\left(s\right)=\frac{V_{O}}{V_{S}}=\frac{Z_{B}//Z_{A}}{Z_{Cloth}+Z_{B}//Z_{A}}$$

where “//” represents the parallel combination of two impedances. Here, Z_A_ corresponds to the input impedance of the operational amplifier, which is given by R_A_//C_A_, while Z_B_ accounts for the impedance resulting from the parallel combination of R_B_//C_B_. Additionally, Z_Cloth_ represents the impedance of the cloth, defined as R_Cloth_//C_Cloth_. Given that ∣Z_A_∣ is significantly larger than ∣Z_B_∣, Z_A_ can be neglected. Consequently, the gain simplifies to:

$$G_{s}\left(s\right)≅\frac{R_{B}+sC_{Cloth}R_{B}R_{Cloth}}{\left(R_{B}+R_{Cloth}\right)+s(0.9C_{B}C_{Cloth})R_{B}R_{Cloth}}$$

The derived gain G_s_(s) exhibits the characteristics of a high-pass filter, with its cutoff frequency influenced by the bias resistor R_B_. Lee et al. conducted frequency response simulations for fabrics such as cotton and polyester, investigating R_B_ values ranging from 100 MΩ to 20 GΩ to determine an appropriate resistance [44]. Their findings indicated that resistances below 1 GΩ attenuate the ECG signal, making them unsuitable for ECG measurement. Based on this, an ultra-high-value grounding resistor R_B_ (1GΩ) was included in the capacitive measurement system. The sensor was installed on the back of a chair, as shown in Figure 1b, to detect the differential signal. The output signal from each electrode was amplified and filtered using a Sallen–Key active filter configured as a 0.5–35 Hz band-pass filter. Due to the high impedance between the capacitive electrodes and the body, the capacitive ECG measurement system is prone to common-mode noise. Moreover, the impedance between each capacitive electrode and the body may vary. Thus, in this study, a driven capacitive ground circuit incorporating an additional third electrode for the negative feedback of the common-mode signal was used to effectively eliminate common-mode noise from the body [45]. For a detailed description of the electrode system, please refer to the cited references.

### 2.2. Experimental Design

**Figure 2:**
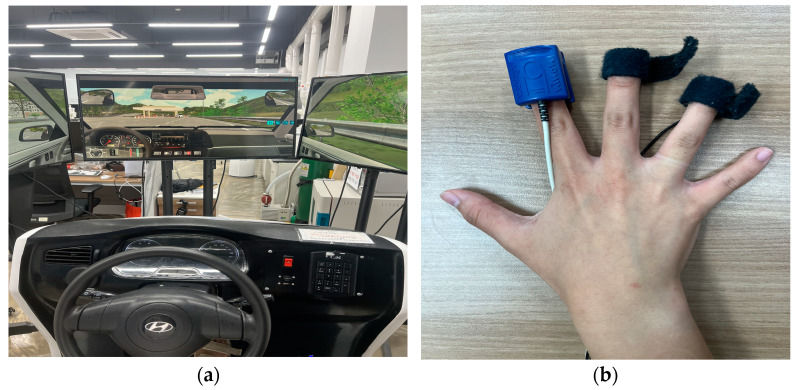
(**a**) Driving simulator (YESA-HW2) and experimental environment and the (**b**) Biopac OXY100E and EDA100C used in the actual experiments.

The study was conducted in accordance with the Declaration of Helsinki. Ten participants (4 males and 6 females; age: 22.4 ± 0.966 years), all holding valid driver’s licenses, took part in this study. All participants were healthy individuals with no diseases that could cause issues with alcohol consumption and were not taking any related medications. This study was approved by the Institutional Review Board (IRB No. 1040875-202211-SB-122) of Soonchunhyang University. Prior to the experiment, participants were fully informed about the purpose and procedures of the study, and their consent was obtained. All participants were asked to refrain from alcohol consumption for 3 days prior to the experiment and to refrain from caffeine consumption on the day of the experiment. Subjects were tested in a driving simulator, as shown in Figure 2a, and sensors for multiple biosignal measurements were attached, as shown in Figure 2b. These sensors were attached to the fingers but are expected to be integrated into the steering wheel in the future to enable unconstrained measurements.

The experiment was divided into three distinct conditions: Experiment A with BrAC: 0.00%, Experiment B with BrAC: 0.03%, and Experiment C with BrAC: 0.08%. These experimental conditions were designed to align with Korea’s driving under the influence (DUI) enforcement standards, ensuring that the BrAC levels in each trial accurately represented legal thresholds. Each experiment lasted approximately 20 min, consisting of two 10 min phases.

**Figure 3:**
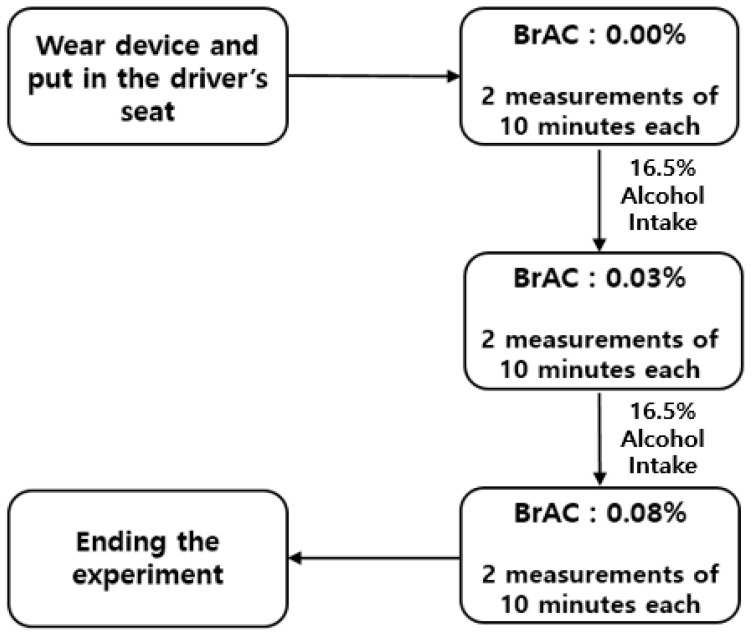
Block diagram of the experimental process.

After completing a 10 min experiment, the participants adjusted to a comfortable posture before continuing with an additional 10 min experiment. This procedure was conducted twice for each condition, resulting in a total data collection period of 60 min. After completing two 10 min experiments without alcohol, the participants consumed alcohol with an alcohol content of 16.5% until the designated breath alcohol concentration (BrAC) was reached. To monitor this, the participants measured their BrAC every 5 min using a breathalyzer (AL9000, Sentech Korea, Inc., Paju, Republic of Korea). When the BrAC reached 0.03%, they performed two 10 min experiments. Similarly, when the BrAC reached 0.08%, two additional 10 min data collections were conducted following the same procedure. In each case, the 20 min duration included a 10 min experiment followed by a brief adjustment to a comfortable posture and an additional 10 min experiment. The entire experimental process is illustrated in Figure 3.

### 2.3. Data Preprocessing

The final dataset consisted of ECG, PPG, and EDA measurements collected from 10 participants. However, due to severe motion artifacts in the data from one participant, their recordings were excluded, leaving a total of nine participants for analysis. For each participant, data were recorded twice for 10 min across three drinking situations. To address the challenges of rapid alcohol detection and limited data availability, the collected data were segmented, and feature extraction was performed. We applied a sliding window method to segment the vital sign data (ECG, PPG, and EDA) into overlapping 30 s segments with a 10 s overlap. This approach increased the dataset size for training a machine learning classifier and facilitated the extraction of additional features.

**Figure 4:**
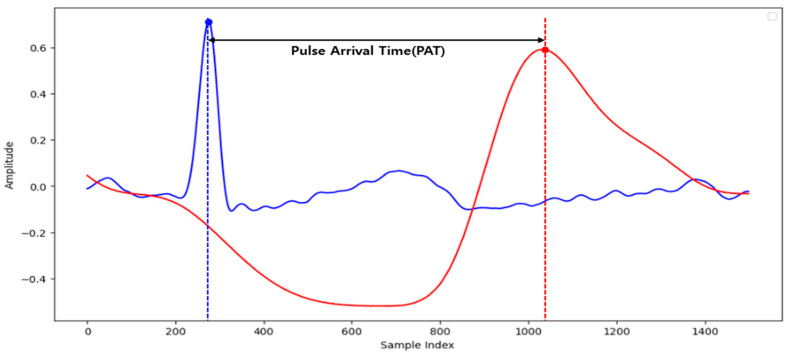
An example of pulse arrival time (PAT) as one of the features of this study, which can be calculated on an hourly basis using the peak of the PPG and the peak of the R-peak of the simultaneously measured ECG.

We analyzed the ECG data to assess HRV, which reflects changes in heartbeat intervals. HRV provides insights into autonomic nervous system activity, indicating a balance between sympathetic and parasympathetic responses. From this analysis, we extracted five frequency domain features, four time-domain features, four Poincaré plot features, and six entropy domain features. Additionally, pulse arrival time (PAT) was extracted from the PPG and ECG data. PAT measures the interval between the R-peak of the ECG signal and the peak of the PPG signal detected in the peripheral blood vessels, serving as an important indicator of cardiovascular status, as illustrated in Figure 4. We calculated the mean PAT (Mean_PAT) and standard deviation of PAT (std_PAT), incorporating these metrics into the feature matrix alongside the HRV results. Furthermore, we extracted the standard deviation of EDA (std_EDA) to evaluate EDA signal variability, which can be influenced by environmental factors and individual baseline differences. The extracted biosignal features are summarized in Table 1. Finally, the data were prepared in the form of a matrix of 23 (feature + label) × 35 (biosignal data every 30 s using a sliding window) × 54 (number of data files).

In this study, we evaluated various machine learning classifiers to identify the most effective model for classifying drivers’ drinking situations based on the extracted ECG, PPG, and EDA features. The classification process was conducted in a Google Colab environment, leveraging its computational resources for efficient model training and testing. The classifiers used included Support Vector Machine (SVM), Random Forest (RF), K-Nearest Neighbors (K-NN), XGBoost, Stochastic Gradient Descent (SGD), Logistic Regression, Gradient Boosting, Multilayer Perceptron (MLP), and Linear Discriminant Analysis (LDA). These models were chosen for their ability to handle complex, high-dimensional data and their effectiveness in classification tasks. Additionally, ensemble methods like Bagging, Voting Classifiers, and Deep Neural Networks (DNNs) were explored to improve model performance and reduce overfitting.

**Figure 5:**
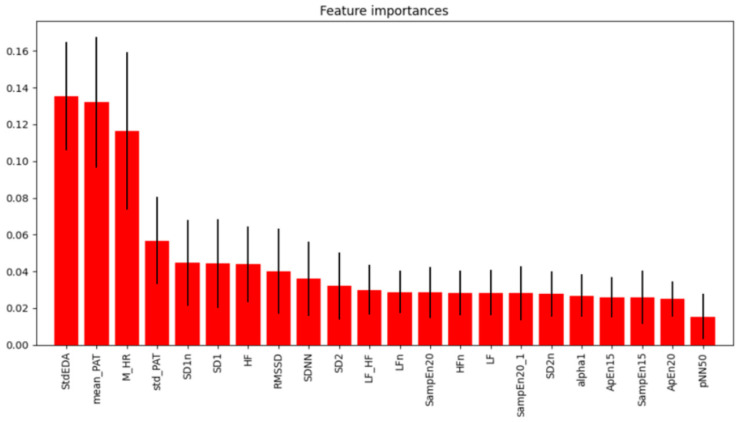
Feature importance from the Random Forest method. The Elbow method chose the top three features with the largest learning impact (up to M_HR) and the top nine features with the next-largest decreasing impact (up to SDNN).

To enhance model performance and reduce computational complexity, we applied a feature importance method based on Random Forest to identify and eliminate non-contributing features. This approach focused on the most impactful variables, as irrelevant features can increase data processing overhead without improving classification accuracy. Additionally, the Elbow method was employed to determine the optimal number of features to retain, helping to identify the point at which additional features no longer significantly enhanced model performance. The results of the feature importance analysis and the application of the Elbow method are shown in Figure 5.

## 3. Results

### 3.1. Analysis of HRV Characteristics and Other Biosignal Change Rates

This study investigated the impact of alcohol consumption on HRV, PAT, and EDA at three levels of BrAC: 0.00%, 0.03%, and 0.08%. These features, derived from ECG, PPG, and EDA data, reflect key physiological changes in the ANS and cardiovascular function associated with alcohol intake. The results reveal distinct patterns of change across the examined BrAC levels, offering insights into the effects of alcohol on physiological processes. The average and standard deviation for each feature at each BrAC level are summarized in Table 2.

HRV metrics, which reflect autonomic control of the heart, exhibited notable alterations with increasing BrAC levels. SDNN, an indicator of overall HRV and the combined influence of sympathetic and parasympathetic activity, decreased from 39.29 ± 17.35 at a BrAC of 0.00% to 32.50 ± 26.61 at 0.08% (*p* = 0.035). This reduction suggests an overall decline in autonomic flexibility. Similarly, pNN50, which quantifies parasympathetic modulation, showed a significant decrease from 5.01 ± 6.55 to 2.47 ± 5.55 (*p* = 0.047), indicating reduced vagal (parasympathetic) activity. The LF_HF ratio, which reflects the balance between sympathetic and parasympathetic influence, increased from 3.87 ± 5.04 to 4.75 ± 5.91 (*p* = 0.041), pointing to a shift toward sympathetic dominance. While RMSSD, another marker of parasympathetic activity, decreased from 30.21 ± 18.34 to 27.24 ± 43.96, the *p*-value (0.067) from paired *t*-tests suggests that this change was not statistically significant. These results indicate that alcohol consumption at higher BrAC levels is associated with reduced parasympathetic modulation and increased sympathetic activity, disrupting normal autonomic equilibrium. In addition to HRV metrics, PAT, which measures vascular dynamics and arterial stiffness, showed significant changes. Mean_PAT increased from 0.33 ± 0.03 s at a BrAC of 0.00% to 0.34 ± 0.04 s at 0.08% (*p* = 0.025), suggesting that alcohol consumption leads to slower pulse wave transmission, possibly due to altered vascular compliance. However, std_PAT changes, which measure variability in PAT, were not statistically significant (*p* = 0.089), indicating that the variability in vascular dynamics was not affected uniformly across subjects. The *p*-values for these changes were also calculated using paired *t*-tests.

**Figure 6:**
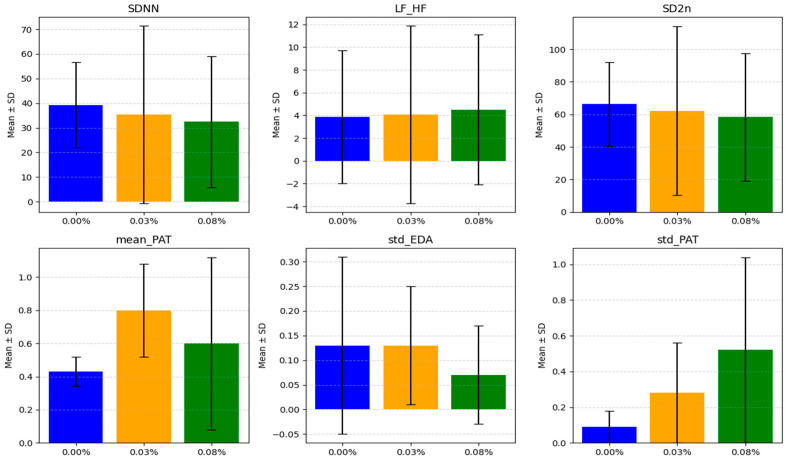
Graphs of means and standard deviations of HRV features (SDNN, LF_HF, SD2n), PAT, and EDA indicators with BrAC level (blue: BrAC: 0.00%, orange: BrAC: 0.03%, green: BrAC: 0.08%).

EDA, which reflects changes in sweat gland activity driven by sympathetic nervous system stimulation, also exhibited significant alterations. The standard deviation of EDA (std_EDA) decreased from 0.13 ± 0.18 at a BrAC of 0.00% to 0.10 ± 0.10 at a BrAC of 0.08% (*p* = 0.049), indicating reduced variability in sympathetic arousal. This reduction may reflect diminished responsiveness of the sweat glands or decreased activation of the sympathetic pathways responsible for controlling EDA. The statistical significance of these changes was confirmed through paired *t*-tests. The means and standard deviations for metrics that increase or decrease linearly with BrAC, PAT, and EDA are shown in Figure 6.

These results demonstrate that alcohol consumption induces several physiological changes that disrupt the normal functioning of the autonomic and cardiovascular systems. Specifically, the decrease in parasympathetic activity, increased sympathetic dominance, slower vascular responses, and reduced variability in sweat gland activity suggest that alcohol suppresses certain physiological regulatory mechanisms while over-activating others. These alterations provide a clear physiological basis for the systemic effects of alcohol intoxication, offering measurable indicators of its impact on the body.

### 3.2. Machine Learning Classification Results

In this study, we explored various machine learning classifiers to determine the most effective model for classifying drunk driving situations (BrAC: 0.00%, BrAC: 0.03%, and BrAC: 0.08) based on ECG, PPG, and EDA features. The classification process was performed in a simple but powerful Python Google Colab (version 1.2.0) environment for AI classification, utilizing computing resources for efficient model training and testing. To improve the performance of the model and reduce the computational complexity, we adopted the feature importance method using a random forest classifier. This approach allowed us to identify and prioritize the most relevant features while removing features that minimally contributed to or interfered with the learning process of the model. Features that did not significantly improve the classification accuracy were removed to simplify the learning process and focus on the variables that had the greatest impact. From the initial 22 biosignal features, we applied the feature importance method followed by the Elbow method to select the most relevant subset of features. Feature importance analysis highlighted the top nine features, and further refinement identified the top three. We used the Elbow method to determine the optimal number of features to retain, selecting a subset of features that maximized the model performance without unnecessary complexity.

The machine learning classifiers used in this study were Support Vector Machine (SVM), Random Forest, K-Nearest Neighbor (KNN), XGBoost, Stochastic Gradient Descent (SGD), Logistic Regression, Gradient Boosting, Neural Network, Naive Bayes, Linear Discriminant Analysis (LDA), Bagging, Voting Classifier, and Deep Neural Network (DNN). The reason for using these different models is to improve the ability to handle complex, high-dimensional data, improve efficiency in classification tasks, and avoid overfitting situations.

Each classifier was evaluated using a dataset generated from the top features identified via feature importance. A grid search was performed to optimize the hyperparameters of each model, and five-fold cross-validation was used to evaluate performance metrics of accuracy, precision, recall, and F1 score.

Through this comprehensive evaluation process, we aimed to identify the classifier and feature set combination that offered the highest level of accuracy and generalizability across varying intoxication states to enhance the reliability of the classification system and provide insights into the physiological markers most closely associated with alcohol consumption. The machine learning results using all features, the top nine features, and the top three features are summarized in Table 3, Table 4 and Table 5, respectively.

## 4. Discussion

This study aimed to develop an effective system for detecting alcohol intoxication in drivers using the non-invasive biometric signals of ECG measured unconstrained using capacitive electrodes, PPG, and EDA. Through feature extraction and machine learning classification, the most relevant features and the best algorithms were identified for classification, improving the accuracy and practicality of alcohol detection in real-world driving scenarios. Among the 22 features extracted, HRV, PAT, and EDA were the most important for distinguishing BrAC. HRV is a well-established biomarker for ANS activity, and it is particularly sensitive to alcohol consumption, which impairs the body’s ability to regulate heart rate. Previous studies have shown that acute alcohol consumption leads to significant reductions in HRV, particularly in time-domain measures like SDNN and RMSSD, which assess overall HRV [46]. These features were highly influential in our study as well, confirming their relevance for BrAC classification.

Pulse arrival time (PAT), which is the time difference between the R-wave peak in the ECG signal and the corresponding peak in the PPG signal, was another crucial feature. Alcohol consumption is known to alter PAT due to its effects on blood pressure and vascular tone, which are influenced by the central nervous system. Studies have demonstrated that alcohol affects vascular reactivity, influencing PAT and supporting its role in blood alcohol detection [47]. The combination of HRV, PAT, and EDA as top features reflects the physiological changes induced by alcohol consumption, validating their strong contributions to BrAC classification. Among the various machine learning algorithms evaluated, XGBoost and Gradient Boosting showed the highest accuracy and F1 score. These models excel at handling complex, non-linear relationships in data and are well-suited for the intricate patterns present in biometric signals. XGBoost is an implementation of gradient boosting that uses decision trees as base learners, iteratively improving them using a gradient-based optimization approach. This method is particularly powerful due to its ability to prevent overfitting through regularization and its ability to scale efficiently with large datasets. XGBoost has become a popular choice for structured data classification tasks due to its robust performance and flexibility in handling various data scenarios. In the context of alcohol detection, where interactions between multiple features such as HRV, PAT, and EDA are complex and non-linear, XGBoost was effective, contributing to its superior performance.

Both XGBoost and Gradient Boosting benefit from advanced techniques such as early stopping, which halts training when performance on the validation set begins to degrade, helping to prevent overfitting and maintaining the generalization of new data. These advantages made both models particularly effective in classifying BrAC levels based on biosignal data. On the other hand, simpler models such as logistic regression or naive Bayes, which rely on linear assumptions, struggled to capture the complex non-linear relationships in the data (the accuracy values for SVM, logistic regression, and NDA in classification using the top three features were 0.48, 0.53, and 0.52, respectively). While computationally efficient, these models were unable to achieve the accuracy and precision of XGBoost and Gradient Boosting, highlighting the importance of choosing the right model for tasks involving complex biosignals. Table 6 compares the results of the method proposed in this paper with previously published methods.

Unlike previous methods, the proposed scheme performs three instead of two classifications based on the penalties for driving under the influence of alcohol in Korea and achieves the highest classification accuracy of 88%. This study did not deal with binary classification problems in earnest, but in the case of normal (BrAC: 0.00%) and strong drinking (BrAC: 0.08%), it showed an accuracy of up to 96.4% for Gradient Boosting. In addition, the proposed method adopts the CCECG method using capacitive electrodes for ECG collection, which reduces the inconvenience of attaching electrodes to human bodies during ECG measurement. This advantage increases the likelihood of application in real-world environments. In future research, PPG and EDA sensors could be integrated into the steering wheel to further increase convenience.

This study was designed as an initial feasibility investigation, and we acknowledge that the sample size of 10 participants, with data from 9 used for analysis, is relatively small. However, unlike other studies, we collected data under three distinct conditions: normal, weak drinking, and strong drinking, which allowed us to capture a wide range of physiological responses. Additionally, by employing a sliding window technique during data analysis, we were able to secure a substantial amount of data despite the limited number of participants. This approach not only enhanced the robustness of our dataset but also ensured that the results were based on comprehensive and contextually diverse information. Despite the small sample size, the consistent patterns observed across participants indicate the reliability of the findings within the study’s scope. Future studies will involve larger sample sizes to enhance generalizability and further investigate the effects of individual differences.

Despite the promising results achieved with XGBoost and Gradient Boosting, potential confounding factors must be considered in future studies. Physiological responses such as exercise, stress, or anxiety can influence the same features used for alcohol detection, leading to false positives or misclassifications. For example, both HRV and EDA can decrease or increase, respectively, due to physical exertion or anxiety and may be mistaken for signs of intoxication. Additionally, other factors such as caffeine consumption, medical drug intake, or heightened states of anxiety could similarly affect physiological signals, further complicating the detection process. To mitigate the impacts of such confounding factors, future work could incorporate additional classifiers or decision rules that account for context, such as activity levels, stress indicators, or external influences like caffeine or medication, alongside biometric data. Furthermore, integrating real-time monitoring systems that track these contextual variables could help improve the robustness and accuracy of alcohol detection models, reducing the likelihood of misclassification due to non-alcohol-related changes in physiological signals. By addressing these diverse variables, a more comprehensive and resilient system can be developed for accurate alcohol detection in various real-world scenarios.

## 5. Conclusions

Drunk driving detection is a way to address the global problem of drunk driving. This research proposes a powerful way to address the limitations of traditional drunk driving enforcement. An electrocardiogram is a vital sign analysis that can reliably assess a person’s heartbeat, but it is inconvenient to apply in daily life. In this study, we collected multiple vital signs according to the Korean DUI penalty standards using capacitive electrodes to collect electrocardiograms using an electrode-free method and PPG and EDA. After extracting 22 features, we found that HRV indicators such as SDNN consistently decreased with increasing alcohol concentration, indicating a decrease in parasympathetic control and a potential increase in sympathetic dominance. Using the feature importance method and 12 machine learning techniques, we found the best model, which achieved a maximum accuracy of 88%. These results suggest that multiple vital signs can be used to predict driver intoxication and could be a powerful way to address the problems of traditional BrAC collection methods in DUI enforcement. Although various methods for biometric-based driver sobriety detection are being developed, our system is innovative in that it is collected in everyday clothing in the driver’s seat without any electrode attachment. We hope that this research will lead to new strategies for driver sobriety detection.

## Figures and Tables

**Table 1:** Features obtained using ECG, PPG, and EDA.

Features	Summary
M_HR	5 min average heart rate
SDNN	Standard deviation of heart rate variability
RMSSD	Root mean square of successive RR interval differences
pNN50	Proportion of RR intervals that differ by more than 50 ms
LF	Power in the low-frequency band
LFn	Relative power in the low-frequency band
HF	Power in the high-frequency band
HFn	Relative power in the high-frequency band
LF_HF	Ratio of low-frequency to high-frequency power
SD1	Measure of short-term heart rate variability
SD2	Measure of long-term heart rate variability
SD1n	SD1 normalized by total variability
SD2n	SD2 normalized by total variability
alpha1	Center frequency of the low-frequency band
ApEn15	Measure of irregularity for 15-point embedding
ApEn20	Measure of irregularity for 20-point embedding
SampEn15	Sample entropy for 15-point data
SampEn20	Sample entropy for 20-point data
SampEn20_1	Sample entropy for 20-point data (version 1)
mean_PAT	Average pulse arrival time
Std_PAT	Standard deviation of pulse arrival time
std_EDA	Standard deviation of skin conductance

**Table 2:** The average and standard deviation for each feature at each BrAC level are summarized. Key metrics such as SDNN, LF_HF, and SD2n reflect altered autonomic control with reduced parasympathetic activity and increased sympathetic dominance. Features in bold are those that had a *p*-value less than 0.1.

Feature	BrAC (0.00%)	BrAC (0.03%)	BrAC (0.08%)
M_HR	80.30 ± 6.31	90.74 ± 9.11	88.46±8.50
SDNN	**39.29 ± 17.35**	**35.38 ± 35.96**	**32.50 ± 26.61**
RMSSD	30.21 ± 18.34	34.37 ± 55.12	27.24 ± 39.06
pNN50	5.10 ± 6.53	2.066 ± 4.06	2.47 ± 5.55
LF	0.001 ± 0.004	0.001 ± 0.003	0.001 ± 0.002
LFn	63.8 ± 21.89	62.94 ± 20.42	66.14 ± 22.11
HF	0.001 ± 0.008	0.001 ± 0.005	0.001 ± 0.003
HFn	36.10 ± 21.89	37.06 ± 20.42	33.86 ± 22.11
LF_HF	**3.87 ± 5.84**	**4.075 ± 7.79**	**4.51 ± 6.58**
SD1	0.02 ± 0.01	0.025 ± 0.04	0.02 ± 0.03
SD2	0.05 ± 0.02	0.042 ± 0.03	0.040 ± 0.03
SD1n	28.08 ± 15.04	36.93 ± 61.71	28.17 ± 43.37
SD2n	**66.31 ± 25.86**	**62.16 ± 51.92**	**58.31 ± 39.22**
alpha1	1.09 ± 0.32	1.10 ± 0.39	1.15 ± 0.40
ApEn15	0.20 ± 0.11	0.280 ± 0.15	0.27 ± 0.16
ApEn20	0.32 ± 0.12	0.39 ± 0.14	0.38 ± 0.16
SampEn15	2.20 ± 0.52	1.90 ± 0.71	1.99 ± 0.65
SampEn20	1.88 ± 0.40	1.64 ± 0.61	1.71 ± 0.57
SampEn20_1	1.88 ± 0.40	1.64 ± 0.61	1.71 ± 0.57
mean_PAT	**0.43 ± 0.17**	**0.80 ± 0.80**	**0.60 ± 0.52**
Std_PAT	0.03 ± 0.09	0.28 ± 0.50	0.16 ± 0.33
std_EDA	**0.13 ± 0.18**	**0.13 ± 0.12**	**0.07 ± 0.10**

**Table 3:** Feature classification. The machine learning model that showed the highest classification performance was the XGBoost model, with an accuracy of 88%. The machine learning model that showed the lowest classification performance was the SVM model, with an accuracy of 51%.

Machine Learning Model	Accuracy	Precision	Recall	F1-Score
SVM	0.51	0.57	0.53	0.5
RF	0.84	0.85	0.85	0.85
K-NN	0.64	0.57	0.57	0.57
XGBoost	0.87	0.89	0.89	0.89
SGD	0.54	0.61	0.58	0.53
Logistic Regression	0.54	0.58	0.56	0.56
Gradient Boosting	0.88	0.88	0.88	0.88
MLP	0.58	0.64	0.64	0.64
LDA	0.53	0.55	0.55	0.55
Bagging	0.86	0.87	0.87	0.87
Voting	0.73	0.73	0.72	0.72
DNN	0.71	0.71	0.70	0.71

**Table 4:** The top nine feature classification models. The machine learning model that showed the highest classification performance was the Gradient Boosting model, with an accuracy of 88%, and the machine learning model that showed the lowest classification performance was the SVM model, with an accuracy of 49%.

Machine Learning Model	Accuracy	Precision	Recall	F1-Score
SVM	0.49	0.55	0.51	0.47
RF	0.87	0.88	0.88	0.88
K-NN	0.71	0.76	0.75	0.75
XGBoost	0.88	0.89	0.88	0.88
SGD	0.5	0.49	0.55	0.47
Logistic Regression	0.53	0.54	0.52	0.51
Gradient Boosting	0.88	0.89	0.89	0.89
MLP	0.56	0.66	0.65	0.65
LDA	0.54	0.56	0.53	0.52
Bagging	0.87	0.87	0.87	0.87
Voting	0.80	0.80	0.80	0.79
DNN	0.78	0.77	0.78	0.77

**Table 5:** The top three feature classification models. The machine learning models that showed the highest classification performance were Random Forest, XGBoost, and Gradient Boosting, each with an accuracy of 81%, and the machine learning model that showed the lowest classification performance was the SVM model, with an accuracy of 48%.

Machine Learning Model	Accuracy	Precision	Recall	F1-Score
SVM	0.48	0.60	0.52	0.48
RF	0.81	0.81	0.81	0.81
K-NN	0.66	0.68	0.68	0.68
XGBoost	0.81	0.79	0.79	0.79
SGD	0.50	0.53	0.54	0.50
Logistic Regression	0.53	0.54	0.53	0.52
Gradient Boosting	0.81	0.80	0.80	0.80
MLP	0.53	0.57	0.57	0.56
LDA	0.52	0.54	0.53	0.51
Bagging	0.81	0.83	0.83	0.83
Voting	0.76	0.77	0.77	0.77
DNN	0.61	0.63	0.61	0.6

**Table 6:** Comparison of the proposed work with previous methods.

Study	Classification Models	Inputs	Number of Classifications	Accuracy
[18]	SVM	ECG, PPG, Car data	2 (Normal, Drunk)	95%
[48]	Weighted Kernel SVM	ECG	2 (Normal, Drunk)	87.52%
Proposed work	12 machine learning methods	CCECG, PPG, EDA	3 (Normal, Light, and Heavy Drunk)	88%

## Data Availability

Data are unavailable due to privacy constraints.
